# On the Internal Use of Chloroform in the Treatment of Delirium Tremens

**Published:** 1866-11

**Authors:** E. M’Clellan

**Affiliations:** Assistant Surgeon, U. S. Army


### On the Internal Use of Chloroform in the Treatment of Delirium Tremens.

BY E. M'CLELLAN, M. D., ASSISTANT SURGEON, U. S. ARMY.

In the treatment of delirium tremens, be it presented either in that stage of excitement of the nervous system, dependent upon the excessive stimulation of a late debauch, or in that of cerebral debility, the result of a total absence of the long accustomed stimulant, the same results must be obtained in order to afford that relief which the condition of the patient demands; and in either case is the condition of the nervous system produced by excessive stimulation alone to be considered.

By the constant presence of alcohol, the stomach has lost its tone, and among those in whom this disease occurs but little food is taken at any time, while during a debauch it is almost entirely abstained from.

Inanition is therefore a serious complication.

During a debauch the drunkard for the time becomes a pedestrian, and, in his sleepless walk, traverses distances which, at other times, are far beyond his powers. He exposes himself to all extremes of temperature; this, with his total neglect of all hygienic laws, swells the account of his folly, until finally, by the nervous excitement operating upon his prostrate system, the vital force of the patient is reduced to that point from which, unaided, he can rarely recuperate.

It is conceded by all, that the first indication in the treatment of this disease is to subdue the undue excitement which prevails, and that this must be accomplished, and sleep procured, before further action is practicable. Therefore, a remedy which will most readily procure this result is the one to be exhibited.

Rarely has there been a requirement of disease to meet which such a variety of remedies have been proposed; they range from the sedative effect of solitary confinement to free stimulation; from active antiphlogistic treatment to that of powerful narcotics; and among others of this class is found *chloroform*, exhibited by inhalation or administered internally.

Frequently has this remedy been recommended for internal use in this disease and others of the same family; its claims have been presented with great earnestness by some observers; it has met with many strong advocates; but the mass of the profession look upon it with distrust, forgetting that they daily, and with impunity, use products far more deadly in their character.

It has been clearly demonstrated that chloroform administered internally acts as a "*diffusible narcotic which is as free from danger as any other drug; that in its somniferous properties it is more prompt than opium, and that its effect is of shorter duration, producing less cerebral oppression.*"

Among the cases recorded of its internal use, the following have been selected, and are referred to, as corroborating this opinion:

I. In the Dublin *Medical Press* for 1852, Mr. Butcher reports in full his treatment of a case of delirium tremens, in which, all other remedies failing, it was employed with perfect success.

II. In the Dublin *Quarterly Journal* for May, 1863, Dr. Harvey reports a case of maniacal delirium, in which it was internally used with great success, opium and other narcotics having failed.

III. In the Dublin *Hospital Gazette* for February, 1854, Dr. Gordon reports its employment with advantage in two cases of violent delirium, the result of irritative fever.

IV. In the same Journal, Dr. McDowell reports its successful administration in three cases of delirium tremens.

These cases have been referred to, not only from the fact that they fully demonstrate the internal action of chloroform, but that they are those in which full physiological doses were administered, and also those in which its effects were not impaired by combinations with other substances.

The rapidity with which chloroform acts upon the nerve centers; the character of the sleep produced; its not producing cerebral depression or gastric irritation, its prompt relief of nausea, and arrestion of vomiting, shorten materially the period during which the practitioner must remain inactive before he can meet those complications which exert so powerful an influence upon this disease.

This remedy has been freely used by the writer, and the appended cases, from the record of those treated, show its uniform action.

CASE I.—Private B. was admitted to the Post Hospital, Fort Delaware, September 12th, 1865. This patient had been drinking hard for several days, and was reported as having no sleep during the past forty-eight hours. He was much debilitated, pulse about 100, skin cold and clammy, tongue heavily coated, very restless, suffering from constant nausea, and very considerable tremor. No mental disorder beyond an inability to concentrate his mind upon any subject. Chloroform ʒj was administered, followed by a small quantity of iced water, and was not ejected, the tremor gradually ceased, his pulse became fuller and less frequent. He was undressed and placed in bed, and shortly passed into a profound sleep, which lasted for nearly five hours, at the expiration of which he awoke, exhibiting no symptoms of the attack beyond that of debility. He remained under treatment until the 14th, when he was returned to duty.

CASE II.—Private M., aged 40 years, originally of a strong constitution, but much debilitated by prolonged attacks of intermittent fever, was arrested, September 29th, 1865, at about 8 o'clock, P. M., and confined, after he had been drinking hard for many hours.

He was violently excited, and much force was necessary before he could be secured; his condition during the night continued the same.

My attention was not called to his case until 8 o'clock A. M., the next day. I found him suffering greatly from prostration, vainly endeavoring to sleep, and pleading for stimulants; muscular tremor was excessive.

Chloroform ʒj was administered, and produced an immediate cessation of his craving for drink.

In twenty minutes, as no permanent relief had been obtained, it was repeated to the extent of half a drachm, and in fifteen minutes the patient was in a sleep which lasted for several hours. When he awoke his mind was clear, and the nervous prostration relieved. He remained under treatment until October 2d, when he was returned to duty.

Case III.—On the morning of June 10th, 1866, was called to see private D., who was in close confinement, and found that he had been drinking excessively for several days, but that during the past twenty-four hours, had been without any stimulant. The patient was lying upon his blankets, to all appearances perfectly quiet and composed; insomnia being at the first glance the only prominent symptom—the excessive excitement having passed off and the remaining nervousness being to a great degree mastered by the will of the patient. His tongue was heavily coated, the pulse was small and feeble. He had voided no urine during the past ten hours, and had taken no food during the time he had been confined.

On being thrown off his guard, and excited, some muscular tremor, with slight incoherence, came on.

He was placed under the influence of one drachm of chloroform, and was soon in a sound sleep which lasted three hours. On awaking, a full dose of castor oil in porter was administered, and in a few minutes he was again asleep, which lasted for one hour, when he voided about one pint of heavily loaded urine. The sleep continued during the day at short intervals, and under careful treatment and diet he rapidly recovered.

Had this case been allowed to remain, unaided for but a few hours, in all probability it would have terminated in maniacal delirium.

CASE IV.—Private M. H., was admitted to hospital, July 6th, 1866. He had been drinking since early on the morning of the 4th instant; the only symptoms exhibited were insomnia and some nervous excitement. Chloroform ʒi was administered, and was followed by a sleep of two hours, after which he awoke, refreshed and complaining of hunger — ate a full breakfast. The relief afforded was so great that at noon of the same day he was allowed to return to his quarters.

July 7th, 7.30 A. M., he was brought to hospital under guard, with the report that his debauch had been renewed with vigor on the preceding day. He now exhibited the full symptoms of *mania a potu.*

His countenance was anxious; his expression wild; the muscular tremor was fully established; his speech was hurried and irrational. Eyes suffused, tongue heavily coated, pulse about 110, thirst excessive, but vomiting violently after drinking, restless, constantly walking around the ward. Was unable to keep him in bed.

8 o'clock, A. M. Chloroform ʒi was administered in small quantities of simple syrup. The vomiting was arrested, but no other relief obtained.

8.20 A. M. The dose was repeated, and sensibly diminished the rate of his pulse and the violence of the tremor.

8.40 A. M. No disposition to sleep being apparent, the same dose was administered, the tremor gradually ceased, the pulse became more natural, and sound sleep came on, which lasted for over two hours—when he awoke, complaining of severe thirst; still incoherent and affected by the tremor, although a sensible abatement had occurred of all the symptoms. The thirst was relieved by pounded ice, and beef tea in small quantities was administered during the day; he continued drowsy and slept at intervals until five o'clock P. M. At seven P. M., after being awake for about two hours, the restlessness and excitement returned. A drachm of chloroform was again administered, and the patient slept during the entire night.

July 8th. Awoke in the morning much refreshed; his mind clear, but complaining of gastric pain and great thirst; in the absence of his nurse, he drank a large quantity of water, which again brought on excessive vomiting, with every prospect of a return of the graver symptoms. Chloroform gtt. ix exhibited, quieting effectually the vomiting.

Ten grains of calomel were administered, followed by a full dose of castor oil in porter; this produced three evacuations.—The patient remained quiet and rational, did not sleep much during the day—beef tea being retained by the stomach. At 8 o'clock P. M., chloroform ʒss was administered, and secured for the patient a comfortable night.

July 9th. This morning the patient awoke rational, the cerebral symptoms having entirely disappeared, but suffering greatly from debility. From this point the case presents nothing of especial interest; the patient, however, having a hard struggle in recuperating—nausea and vomiting frequently being present, but always yielding to the influence of chloroform.—*Medical and Surgical Reporter, August,* 1866.

